# The Current Health Belief of Exercise Conditions of Chinese College Students and Ways of Improvements: An Analysis Based on the Health Belief Model

**DOI:** 10.3389/fpsyg.2022.906297

**Published:** 2022-07-22

**Authors:** Lamei Gong, Jiazhi Sheng

**Affiliations:** ^1^Laboratory of Sports and Health Promotion, School of Physical Education, Sichuan University of Arts and Science, Dazhou, China; ^2^Graduate School of Management, Management and Science University, Shah Alam, Malaysia

**Keywords:** perceived benefits, perceived subjective barriers, perceived objective barriers, perceived severity, self-efficacy of exercise, cues to action

## Abstract

The purpose of this study was to discuss the differences in the parameters of exercise health beliefs among college students of different genders, and assess the relationship between demographic factors and parameters of exercise health beliefs, and examine the relationship between exercise self-efficacy and internal components of exercise health beliefs. A total of 313 Chinese college students from the Sichuan University of Arts and Science completed the exercise health belief scale voluntarily under the tutor’s explanation. The results showed that compared with female students, male students have higher perceived benefits and self-efficacy of exercise and lower perceived subjective and objective barriers. Monthly family income has a significant positive correlation with exercise self-efficacy and a negative correlation with perceived subjective barriers to exercise disorder. Exercise self-efficacy has a positively correlated with perceived benefits and perceived severity and a significant negative correlation with perceived subjective and objective barriers. From the structural equation model, we found that family income no longer significantly impacted exercise self-efficacy. Within the exercise health belief items, we found only that there were negative relationship between perceived subjective barriers and exercise self-efficacy. According to the study, our findings provide a new psychological angle for understanding the exercise condition of college students and the restraining factors and provide new insights into increasing exercise self-efficacy to lower the subjective barriers to exercise. Future studies will focus on evaluating the relationship between exercise health belief components and college Students’ physical activity levels and exercise behaviors.

## Introduction

In recent years, obesity and a lack of physical exercise have caused a decline in physical fitness among Chinese residents, which has led to a continuous increase in the prevalence of chronic diseases (Tian et al., 2016). The current situation of college Students’ physical exercise is not optimistic. One survey of college students from Shanghai (China) reported by Yajie (2019) found those female college students principally exercised in running and badminton. The frequency of exercise was 1–2 times per week, 20–59 min per time, which is significantly lower than the World Health Organization’s recommendation for physical activity. The guidelines described in this recommendation showed that a person needs to participate in no less than 30 minutes of moderate-intensity exercise each time and at least five times per week in order to maintain their current health. Another study on the current situation of college Students’ physical exercise in China shows that only 19.9% of the surveyed subjects exercise regularly (Lyu et al., 2022), with a lower rate of excellence in physical fitness tests. Similar health-related problems have become an increasingly serious concern for modern Chinese university students. It is worth noting that the physical fitness of college students has continued to decrease over the years. According to the available reports, the obesity rate and overweight rate among Chinese college students are far beyond the excellence rate of physical fitness. A cross-sectional investigation on the relationship between physical exercise, physical fitness tests and health related risks conducted among 1,500 students from Tsinghua University (Wang, 2019) found that students who were inadequate in the exercise had a 1.25 times higher risk of developing obesity than those who regularly participated in the exercise. As a simple index, the BMI-body mass index, which is used to assess the nutritional status of persons or groups, is widely applied in the public health field (Finucane et al., 2011; Elagizi et al., 2018). Scholars have also discussed the relationship between the BMI of Chinese college students and physical fitness. The results elaborated that the higher in BMI, the lower in score on physical fitness, which suggests the importance of HBM interference and physical exercise in health education and health promotion (Chang and Chen, 2015; Chen et al., 2020). Based on the above studies, it can be found that the main factor leading to insufficient physical activity of Chinese college students is lower health beliefs.

The health belief model (HBM) comprises perceived susceptibility to disease, perceived benefits, perceived barriers, perceived severity, cues to action, and self-efficacy. It is one of the behavioral health theories that have been most ancient and most widely applied (Glanz et al., 1991, 2008; Hochbaum et al., 1992). The theory systematically studies the origin of behavioral health theories (Hochbaum et al., 1992; Kharrazi et al., 2009) and has the function of identification, explaining and predicting, and preventing of health behaviors (Janz and Becker, 1984). Since it developed, the HBM has been applied in various aspects of the public environment, such as helping to increase the voluntary participation rate in cervical cancer screening (Hay et al., 2003) and the breast cancer self-check rate (Umeh and Rogan-Gibson, 2001).

According to recent studies, the composite variables of the HBM are capable of predicting BMI or correlating with weight reduction behavior (McArthur et al., 2018). It embodied that weight reduction behaviors significantly correlate with a perceived threat, perceived self-efficacy, and cues to action. For the underweight group, perceived threat and self-efficacy were the significant, influential factors. However, cues to action are essential variables in the overweight group. Perceived severity, susceptibility, perceived barriers, and benefits predict BMI, which can be the foundation of preventative measures relevant to weight, solve specific problems of college students, and meet their needs and objectives. HBM, as a framework capable of predicting and explaining physical activity (Rahmati-Najarkolaei et al., 2015), it was not only used to evaluate the physical activity status of the different aged groups (Gristwood, 2011), and women of pregnancy (Shafieian and Kazemi, 2017), but also investigate the physical activity of different middle-aged groups of women with various cultural backgrounds (Hosseini et al., 2017). Self-efficacy and perceived barriers were the two most significant predictors of or explained physical activity levels in the health belief model. Previous studies have elaborated that older adults with higher exercise self-efficacy remain physically active at the 9th, and 12th months after the end of a strength training intervention (Neupert et al., 2009). Even in patients who suffered from coronary heart disease, self-efficacy was a significant moderator of short-duration exercise behavior during the 1-year follow-up after discharge (Slovinec et al., 2014). A cross-sectional survey of postmenopausal Australian women found that schedule conflict, difficulty getting to physical exercise locations, and bad weather were the dominating barriers preventing them from exercising and that women who exercised regularly had higher levels of self-efficacy (Barnett and Spinks, 2007). Another study reported by Simonavice and Wiggins (2008); the survey showed that infrequently exercising students had higher perceived exercise barriers and lower exercise self-efficacy than regularly exercising college students in a mid-southern university in the United States. A study of participants from universities in Shanghai city (China) found that general self-efficacy significantly positively correlated with the intensity, frequency, and duration of physical exercise among female college students (Yajie, 2019). However, the above-related research in view of the health belief model in the field of sports science lacks direct evidence of the exercise health belief model. It needs especially to develop the exercise health belief model (EHBM) scale based on the HBM.

## Exercise Health Belief Model

Currently, HBM comprises 6 key aspects (Rahmati-Najarkolaei et al., 2015). The perceived benefits will effect the individual adoption of exercise or the possibility of keeping exercise. More benefits gained will arouse more aspirations to participate in the training. Although the perceived benefits of exercise will promote the participation of exercise of the individual, perceived barriers (such as inconvenience for the participation of exercise or lack of time and companionship) will hold back the plan for exercise to some degree or affect the execution of the exercise plan. A previous study showed that education and household income positively correlated with perceived benefits (Sosa et al., 2021). The exercise self-efficacy is the confidence in one’s exercising capability. The self-efficacy of exercise is an essential predictive index for adopting and maintaining exercise behavior. Self-efficacy is a belief and faith; a person can execute the given activity successfully (Fletcher and Banasik, 2001). It can accurately predict the overall exercise level of adult sedentary females who sit for a long time during the day (McAuley and Jacobson, 1991). The self-efficacy of females and males is different due to biological, social, and cultural factors. It significantly increases the physical activeness level of Chinese immigrant females (Cho and Lee, 2013). Currently, available literature has shown that an increase in exercise levels can improve the self-efficacy of exercise (Darawad et al., 2016, 2018; Daniali et al., 2017; Colangelo and Weissbrod, 2019; Kooijmans et al., 2020). However, the correlation between the self-efficacy of exercise and the other factors of the HBM remains unknown. Cues to action encourage individuals to take healthy actions. If a person decides to act on his exercise plan, first of all, he has realized that inadequate exercises would cause health problems. Unless taking exercise would cause health dangers to him, otherwise he will participate in exercises (Biddle and Nigg, 2000). Perceived disease susceptibility involves to a person’s susceptibility to the likelihood that a behavior will harm his or her health or disease. Susceptibility to lack of exercise refers to the possibility of susceptibility to disease due to lack of exercise. Basis the previous study, perceived benefits, perceived severity, perceived barriers, cues to action, and self-efficacy will have an impact on exercise behaviors over time (Dailey, 2001).

Previous studies have explained the factors that influence physical activities, and it is understood that the decisive factors of non-participation in exercises help make interference measures for health problems caused by inadequate exercise. The advantage of the HBM is that it fully considers the influence of social-psychological factors on behavior and uses attitudes and beliefs to explain better and predict behavior. However, HBM is self-limited in that the model does not explicitly point out the relationships between the constituent elements. Some studies have developed the HBM for measuring the relationship between the HBM and disease or behaviors. In contrast, no available examinations have been conducted on the exercise health beliefs of college students. Hence, this investigation aimed to discuss the exercise health beliefs of college students and the influential factors of the self-efficacy of exercise.

The self-efficacy of exercise (Darawad et al., 2016, 2018; Kooijmans et al., 2020) and previous studies have reported barriers and benefits (Darawad et al., 2016). Although previous studies (Wu et al., 2020) developed a new exercise health belief scale, improvements in the self-efficacy of exercise indexes have yet to be found. This study will be different from the former ones in that the self-efficacy of exercise-relevant entries (Park, 2011) has been added to the scale developed by Wu et al. (2020). Therefore, based on previous research, the exercise health belief model contains six items (Dailey, 2001; Wu et al., 2020), including perceived benefit, subjective barriers, objective barriers, severity, cues to action, and exercise self-efficacy. Putting distinguishing perceived barriers as objective and subjective barriers (Wu et al., 2020), this method can effectively help determine the source of the barriers. This study will evaluate the differences in exercise health belief parameters between male and female college students and assess the association among demographic factors, the exercise health belief internal items, and exercise self-efficacy. Therefore, we propose the following three hypotheses:

Hypothesis 1: There are differences in the exercise health belief parameters of college students of various genders.

Hypothesis 2: A significant correlation exists between household income, education level, and perceived benefit and self-efficacy.

Hypothesis 3: There is a correlation between the components of exercise health beliefs and exercise self-efficacy.

## Participants and Methods

### Participants

The study applied the simple random sampling method to select 350 college students from a public college in Western China who completed the test voluntarily. In this study, the investigation was carried out during the outbreak of COVID-19. The specific period is the whole of May 2021. We conducted a face-to-face questionnaire survey. The stand of inclusion and exclusion for the screening sample for this study were as follows: (1) full-time college students, (2) no any physical disability or movement disorder, and (3) no mental disease or disorder. A total of 37 questionnaires were excluded due to incomplete questionnaires (including 15 respondents who voluntarily withdrew from the survey, eight questionnaires lacking gender information, and 14 questionnaires lacking household monthly income information). Finally, a total of 313 valid questionnaires remained, with an effective rate of 89.4%. The students completed the survey form after a detailed explanation from the tutor.

### Ethical Statement

This investigation was approved by the Human Experimental Ethics Board of Sichuan University of Arts and Science (Approval no. 2022SASULL—001) according to the Declaration of Helsinki. All the subjects from the study signed informed-consent forms before completing the survey. They voluntarily participated in the questionnaire survey. All the data collected were processed anonymously and were for research purposes only.

### Study Design

Step 1: Establish and complete the exercise health belief scale, recruit the participants and conduct a survey regarding demography and health belief for exercise.
Step 2: By evaluating the validity and reliability of the questionnaire to determine the final questionnaire items for inclusion in the study.
Step 3: An analysis of demographic and exercise health beliefs differences among male and female college students.
Step 4: Analyze the relationship between demographic information and the construction of exercise health beliefs.
Step 5: Building a Structural Equation Model for assessing factors affecting exercise self-efficacy influencing exercise self-efficacy.

### General Demographic Information Survey

The age, educational level of the participants, and their parents and family monthly income were included and were assigned points. A total of 1–4 points were assigned to educate under elementary education, high school or technical education, graduate degree, and master’s degree. Points for family income (in RMB: Yuan/month): 1.2,000–2,999 (1 score), 2.3,000–3,999 (2 score), 3.4,000–4,999 (3 score), 4.5,000–5,999 (4 score), 5.6,000–6,999 (5 score) 6.7,000–7,999 (6 score), 7.8,000–8,999 (7 score), 8.9,000–10,000 (8 score), 9. more than 10,000 (9 score) (refer Table 1 for survey results).

**TABLE 1:** Descriptive statistics of the participants (mean ± SD).

Items	Overall (*n* = 313)	Male (*n* = 138)	Female (*n* = 175)
Age (years)	19.5 ± 1.5	19.3 ± 1.8	19.7 ± 1.2*
Educational level of students	2.8 ± 0.4	3.0 ± 0.2	2.7 ± 0.5***
Educational level of father	1.2 ± 0.5	1.3 ± 0.6	1.0 ± 0.2***
Educational level of mother	1.1 ± 0.3	1.2 ± 0.5	1.0 ± 0.1**
Family income (Yuan/month)	3.8 ± 2.7	4.9 ± 2.9	2.9 ± 2.1**

*P < 0.05, **P < 0.01, ***P < 0.001.

### Exercise Health Belief Survey

The new scale comprises perceived benefits, subjective and objective barriers, perceived severity, cues to action, and exercise self-efficacy. Among them, perceived benefits have three items; perceived severity has three items, cues to action have three items, and self-efficacy of exercise has 14 items (Park, 2011; Wu et al., 2020). This study restructured the questionnaire design based on previous research literature. Mainly consists of perceived benefits, consisting of a three-question test (e.g., “I think that the reasonable amount of per day is good for keeping health”), perceived subjective barriers, consisting of a three-question test (e.g., “I’m so lazy that not want to exercise”), perceived objective barriers, constituted by a three-question test (e.g., “I have lack of time to exercise?”), perceived severity, by the three-question test, consists of the three-question test (e.g., “I lack energy due to lack of exercise”), and cues to action, which includes the three-question test (e.g., “My friends always remind me to stick to physical activity”) (Wu et al., 2020), and exercise self-efficacy, consists of a 14-question test (e.g., “I’m believe that I can take exercise every day”) (Park, 2011; Wu et al., 2020). A five-point Likert scale assesses each entry (1, strongly disagree; 5, strongly agree).

### Questionnaire Design and Reliability and Validity Test

According to experience, the first step is to examine the validity of the questionnaire. After inspection, the validity coefficient of the scale in this study was 0.821, and *P* < 0.001, which illustrated that the scale has good validity. Cronbach’s alpha values were used for assessing the reliability of the scale. Previous research elaborated that if the Cronbach’s alpha coefficients of the subscale are less than 0.6, it should be considered deleted (Bagozzi and Yi, 1988). Based on preliminary results, we found that the first item of perceived objective barriers, the third item of subjective barriers, and the fifth to fourteenth items of self-efficacy for exercise were deleted. Finally, the final version included 17 items loaded on six factors: perceived benefits (three items), perceived objective barriers (two items), perceived subjective barriers (two items), cues to action (three items), perceived severity (three items), and self-efficacy of exercise (four items).

The six dimensions of the full scale had the following Cronbach’s α values: perceived benefits, 0.825; perceived objective barriers, 0.791; perceived subjectivity barriers, 0.777; perceived severity, 0.560; cues to action, 0.769; and self-efficacy, 0.797 (see Table 2 for survey results). A higher Cronbach’s α value does not always represent higher reliability because the value is influenced by the length of the measured item. If the test item is too short, the value is reduced (Streiner, 2003; Tavakol and Dennick, 2011). Internal consistency is necessary but not sufficient for measuring consistency and unidimensionality (Cortina, 1993). Therefore, we believe retaining the perceived severity is essential based on a previous theoretical framework. The above research illustrated that the scale had acceptable validity and reliability (Sheng et al., 2016).

**TABLE 2:** Reliability coefficients of subscales (Cronbach’s alpha coefficient).

Subscales	Number of items	Items	Cronbach’s alpha coefficient if Item Deleted	Cronbach’s alpha coefficient	Cronbach’s alpha coefficient of subscale if some items deleted
Ben	3	Ben 1	0.824	0.825	
		Ben 2	0.885		
		Ben 3	0.839		
Oba	3	Oba 1*	0.193	0.698	0.791
		Oba 2	0.724		
		Oba 3	0.743		
Sba	3	Sba 1	0.768	0.696	0.777
		Sba 2	0.769		
		Sba 3*	0.394		
Sev	3	Sev 1	0.626	0.560	
		Sev 2	0.768		
		Sev 3	0.663		
Cues	3	Cues 1	0.795	0.769	
		Cues 2	0.822		
		Cues 3	0.845		
Eff-e	14	Eff-e 1	0.776	0.870	0.797
		Eff-e 2	0.764		
		Eff-e 3	0.646		
		Eff-e 4	0.681		
		Eff-e 5*	0.231		
		Eff-e 6*	0.261		
		Eff-e 7*	0.120		
		Eff-e 8*	0.090		
		Eff-e 9*	0.323		
		Eff-e 10*	0.444		
		Eff-e 11*	0.365		
		Eff-e 12*	0.222		
		Eff-e 13*	0.143		
		Eff-e 14*	0.155		

*Item was deleted finally. ben, perceived benefits; oba, perceived objective barriers; sba, perceived subjective barriers; sev, perceived severity; cues, cues to action; eff-e, exercise self-efficacy.

### Data Analysis

The social statistics software resource packages SPSS 23.0 and Amos 23.0 were used for data analysis. Descriptive statistics were used to overview the demographic statistics of the sample and the exercise health belief condition. An independent sample *t*-test was used for evaluating the difference in demographic characteristics, perceived benefits, perceived subjective barriers, perceived objective barriers, perceived severity, self-efficacy of exercise, and cues to action of college students of different genders. In the meantime, the Pearson correlation coefficient was used for describing the relation among demographics and exercise health beliefs and the association between exercise self-efficacy and other factors. In addition, structural equation modeling was used for assessing the relationship among perceived benefits, perceived subjective barriers, objective barriers, perceived severity, cues to action, and exercise self-efficacy. Statistical indexes included minimum discrepancy per degree of freedom (CMIN/DF) and root mean square error of approximation (RMSEA) for construct validity. The statistical significance was set at a *P*-value < 0.05.

## Results

### Analysis of the Current Status of College Exercise Health Belief

**FIGURE 1:**
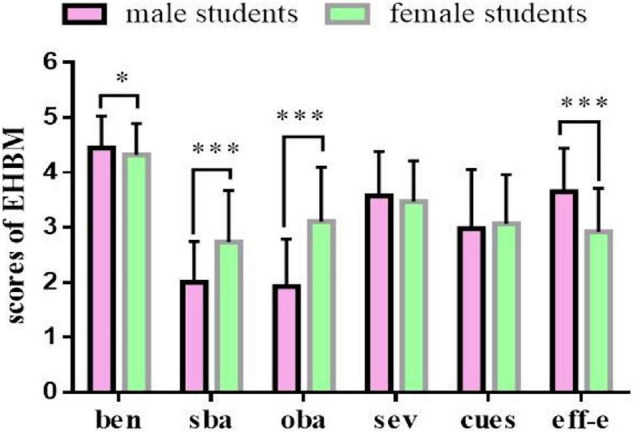
Compared with female college students, male students had higher in perceived benefits and self-efficacy, and lower in perceived subjective barriers and objective barriers. ben, perceived benefits; sba, perceived subjective barriers; oba, perceived objective barriers; sev, perceived severity; cues, cues to action; eff-e, exercise self-efficacy. *p < 0.05, ^***^p < 0.001.

From the current status-analysis of college exercise health belief, compared with female students, male students have higher perceived benefits and self-efficacy of exercise and lower perceived subjective and objective barriers. However, there is no significant difference in perceived severity or cues to action. The above statement responds to hypothesis 1 (refer Figure 1 for details).

### Influential Factors of the Current Status of Exercise Health Belief

From the analysis of influential factors of the current status of exercise health belief, there was a significant negative correlation between the educational level of the Student’s father, monthly family income, and perceived subjective barriers and a significantly positive relationship between monthly family income and self-efficacy of exercise. However, there were no significantly associated between demographic factors and perceived benefits, perceived objective barriers, perceived severity, or cues to action. The above statement responds to hypothesis 2 (refer Table 3 for details).

**TABLE 3:** The relationship between demographic factors and exercise health beliefs (*n* = 313).

Items	Age (years)	Ed-s	Ed-f	Ed-m	income
Ben	−0.04 (0.45)	−0.04 (0.49)	0.11 (0.06)	0.10 (0.08)	0.09 (0.10)
Sba	−0.06 (0.29)	0.07 (0.21)	−**0.14 (0.01)**	−0.09(0.11)	−**0.14 (0.01)**
Oba	−0.02 (0.68)	−**0.161 (0.00)**	−0.08 (0.18)	−0.09(0.11)	−**0.21 (0.00)**
Sev	0.06 (0.29)	0.06 (0.32)	−0.00 (0.94)	0.02 (0.72)	0.00 (0.94)
Cues	−0.00 (0.87)	−0.04 (0.46)	0.09 (0.11)	0.10 (0.07)	0.05 (0.38)
Eff-e	−0.07 (0.20)	**0.13 (0.03)**	0.06 (0.33)	0.09 (0.11)	**0.14 (0.01)**

*Ed-s, educational level of students; Ed-f, educational level of a father; Ed-m, educational level of a mother; income: family income; ben, perceived benefits; oba, perceived objective barriers; sba, perceived subjective barriers; sev, perceived severity; cues, cues to action; eff-e, exercise self-efficacy.*

*Bold values are significant relationship between two variables.*

### Correlational Analysis of the Constructive Exercise Health Belief Factors

From the correlational analysis of the constructive factors of health belief of exercise, self-efficacy of exercise has a significantly positive association with perceived benefits and perceived severity, and it is significantly negatively correlated with perceived objective barriers and perceived subjective barriers. Perceived benefits and perceived severity have a significant negative correlation with perceived objective barriers and perceived subjective barriers (refer Table 4 for details). It is noteworthy that perceived subjective barriers and exercise self-efficacy were negatively correlated (*r* = −0.60, *P* = 0.00).

**TABLE 4:** The relationship between the variables of exercise health belief (*n* = 313).

Items	Ben	Oba	Sba	Sev	Cues	Eff-e
Ben	−	−0.21 (0.00)	−0.15 (0.01)	0.03 (0.55)	0.05 (0.40)	**0.12 (0.03)**
Oba		−	0.61 (0.00)	−0.14 (0.01)	0.05 (0.37)	−**0.50 (0.00)**
Sba			−	−0.21 (0.00)	0.08 (0.17)	−**0.60 (0.00)**
Sev				−	0.19 (0.00)	**0.22 (0.00)**
Cues					−	−0.07 (0.22)
Eff-e						−

*Ben, perceived benefits; oba, perceived objective barriers; sba, perceived subjective barriers; sev, perceived severity; cues, cues to action; eff-e, exercise self-efficacy.*

*Bold values are significant relationship between other variables and self-efficacy.*

### Structural Equation Model Analysis of the Influence of Exercise Self-Efficacy

**FIGURE 2:**
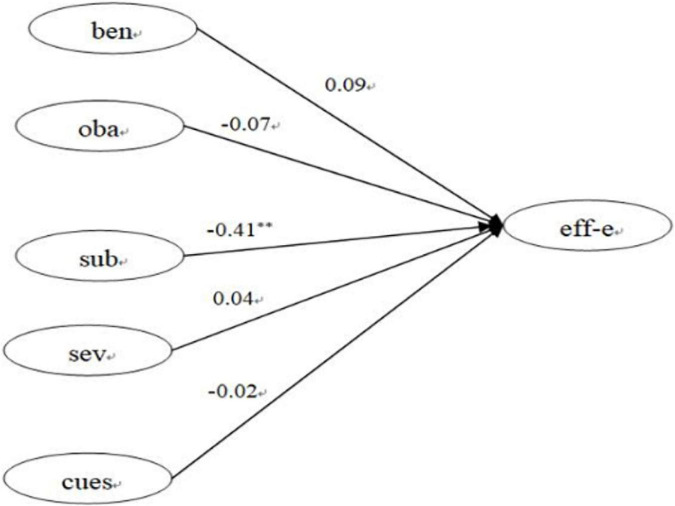
The confirmatory factor analysis. Model—fit indices were statistically acceptable [CMID/DF = 2.781, RMSEA = 0.075, *p* = 0.001]. ben, perceived benefits; oba, perceived objective barriers; sub, perceived subjective barriers; sev, perceived severity; cues, cues to action; eff-f, self-efficacy of exercise. ^**^p < 0.01.

Structural equation modeling is widely used in the social sciences to evaluate the association between social support. The use of mobile programs and physical activity (Wang et al., 2019) assesses physical activity in Turkish schoolchildren (Mirzayi et al., 2021). It identifies determinants of Student Loyalty in Higher Education (Todea et al., 2022). To further explore the relationship between other items in the exercise health belief model and self-efficacy of exercise, we used confirmatory factor analysis with structural equation modeling. In this model, we found only perceived subjective barriers significantly negatively correlated with exercise self-efficacy. No significant correlations were found between perceived benefits, perceived objective barriers, perceived severity, cues to action, and exercise self-efficacy. The above statement responds to hypothesis 3 (refer Figure 2 for details).

## Discussion

### Differences Between Male and Female College Students in Exercise Health Belief Components

The study found that compared with the female students, the male students had lower scores on perceived subjective and objective barriers, with higher scores on perceived benefits and self-efficacy of exercise. According to the various study designs based on the HBM, perceived barriers were proven to be the most influential dimension (Janz and Becker, 1984), which was the most effective predictive factor for young women to self-check their breasts (Umeh and Rogan-Gibson, 2001). Even for older women with colorectal cancer, self-efficacy, doctor referral, perceived benefits, and barriers to screening are important indexes that influence obedience in their participation in screening (Hay et al., 2003). Perceived barriers significantly predict the intention of older adults to participate in physical activities in the community with a history of falling and no such finding among older adults without this history (Kaushal et al., 2021). That may be why the latter group can move around better. This study found that male students had lower scores on perceived barriers and higher perceived benefits and self-efficacy of exercise. This result is consistent with previous reports (Hagger et al., 2001; Klomsten, 2006). The difference in perceived barriers and self-efficacy of exercise between the male and female students may be because of the cognitive tradition based on their different physical characteristics (Klomsten, 2006) and the fact that male students receive more successful experiences from physical exercises (Hagger et al., 2001). In addition, there are some interesting observations from the exercise practice. Female physical education (PE) teachers are more prevalent among male middle school students, and male PE teachers are more prevalent among female middle school students. This study has provided a critical perspective that the school administrator must pay equal attention to the student-teacher relationship and the PE course curriculum setting (Cruz et al., 2021).

### Impact of Demographic Factors on Exercise Health Belief

Self-efficacy and perceived barriers were the important factors of exercise belief that impact exercise behavior. They were more than an essential topic for health education but a strong field of study in sports science. They can be considered a vital factor for predicting exercise behavior (Fletcher and Banasik, 2001). Previous studies believed that factors that impact self-efficacy included parents’ education level (Fletcher and Banasik, 2001; Almutary and Tayyib, 2020) or their academic level and family income level (Sosa et al., 2021). Our study found a significant-association between monthly household income level and perceived self-efficacy, which is consistent with the study of Sosa et al. (2021); at the same time, Sosa et al. (2021) also described a significant positive-association between household income and perceived benefits. However, no such association was found in our study, possibly due to the different ages and cultural backgrounds of the participants. It implied that demographic/environmental factors contributed more to explaining physical activity level than the influential external factors. In addition, this study did not discover other factors that could influence the self-efficacy of exercise and health beliefs of Chinese college students in addition to the factors mentioned above. The probable reason could be that college students are independent in thoughts and behaviors, and the influence of family factors is reduced.

### Exercise Self-Efficacy and Perceived Subjective Barriers

One of the limitations of the HBM is that it is unable to analyze adequately the association between its internal components. In this research, perceived barriers included subjective and objective barriers, which was consistent with Wu’s research report (Wu et al., 2020). This study further reports the relationship between subjective barriers and self-efficacy of exercise. By constructing a structural equation model, we found only a negative association between self-efficacy of exercise and perceived subjective barriers. However, there was no significant association among exercise self-efficacy and perceived benefits, perceived objective barriers, perceived severity, or cues to action. A previous investigation (Shen and Xu, 2008) studied the measurement relationship between self-efficacy and exercise intention of 208 Chinese college students. It found that weight was the only crucial predictive factor relevant to physical activity, while it had no impact on exercise intention. Barnett and Spinks (2007) conducted a cross-sectional survey on 101 postmenopausal females living in North Queensland, located in the tropical zone. He found that the group that had excised for at least 150 min in the past 7 days at medium intensity had a higher exercise self-efficacy and that females with regular exercise were more confident in facing barriers than those with sedentary peers. Their level of perception of obstacles was significantly lower than the latter. This implied that future interference plans should focus on increasing exercise self-efficacy and clearing away barriers so that menopausal women would benefit from physical exercise. Other researchers (Darawad et al., 2016) performed a cross-section investigation on 402 overweight patients from Jordan with chronic diseases. They found that the promise to execute the exercise plan was weakly correlated with barriers and benefits, while no significant correlation was found between self-efficacy and other variables.

Perceived barriers included subjective barriers and objective barriers. This study further reports the relationship between subjective barriers and self-efficacy of exercise. Almutary and Tayyib (2020) further evaluated exercise self-efficacy in chronic patients. The cross-sectional survey found that a lower educational level was the main factor that restrained exercise self-efficacy. That implied that more investigations are needed to assess the impact of perceived benefits and perceived barriers and thus propose interference measures to increase exercise efficacy. Lack of motivation for exercise is a common barrier for older adults with coronary heart disease or diabetes. A study found that older male adults with a better sense of self-efficacy at baseline maintained higher self-efficacy at the 4th and 12th months (Shafieian and Kazemi, 2017). From examining the self-efficacy of exercise and exercise behavior during the strength exercise and after the older adults, which was reported by Neupert et al. (2009), the literature said that the participants who have better physical resilience and exercise effect are relevant with better controlling capacity. Those with better exercise self-efficacy and better ability to control belief at the 6th month persisted in training after the 9th and 12th months. A previous literature (Slovinec et al., 2014) described a longitudinal survey of 801 patients hospitalized due to coronary and heart disease, evaluated their change in exercise behavior, and discussed the key factors that lead to persisting exercise. After 1 year, it was found that motive autonomy and self-efficacy are essential modulation factors in short-term exercise behavior; however, after 6 months, only autonomous motivation is a predictor of long-term exercise behavior.

This survey found a significant association between exercise self-efficacy and perceived subjective barriers. However, there was no significant association among exercise self-efficacy and perceived benefits, perceived objective barriers, perceived severity, or cues to action. Other scholars (Simonavice and Wiggins, 2008) evaluated students at different stages of exercise dependence and evaluated self-efficacy and exercise barriers differently. The literature found that compared with people who were limited to physical exercise or sedentary groups, people who exercise frequently sensed lower exercise barriers and had higher exercise self-efficacy. Higgins et al. (2014) conducted a systematic review and meta-analysis of relevant studies. They found that long-term exercise intervention lowered barriers to self-efficacy, which helped maintain exercise behavior. Dishman et al. (2019) evaluated 79 boys and 108 girls from the United States in grades 5, 6, 7, 9, and 11 with a potential growth model for longitudinal tests. A wearable GT3X (United States) accelerometer was used for physical activity level evaluation. They found that the students who experienced a more significant self-efficacy decrease had a remarkable decline in their physical activity level, and those students experienced higher cognitive barriers to exercise. The enjoyment and fitness goals decreased significantly as well. These results indicated that reducing perceived exercise barriers and maintaining a positive attitude toward the exercise environment is conducive to increasing physical activity.

### Limitations and Future Possibilities

The study surveyed the current status of exercise health beliefs of Chinese college students and found that the most critical factor that restrains the self-efficacy in the exercise was perceived subjective barriers. However, the following limitations still exist. First, the study locked out surveying college Students’ height, weight, and BMI, so it could not discuss the relationship between BMI and exercise health belief construction factors. Future studies will supplement these data. Second, as a cross-sectional survey, this study could not provide conclusive evidence for causal relationships, so future studies will focus on longitudinal tracking research and further assess the influence of additional health education on health beliefs about exercise. Third, the data collected in the survey were the current status of exercise health beliefs of college students from a public college in western China. It was difficult for the study results to represent China’s entire college student group. Future studies will consider more geographical location factors (East vs. West city of China) and school attributes (Private vs. Public) and take a sufficient sample. Finally, this study did not investigate college Students’ physical activity levels or physical exercise behaviors, so it was impossible to assess the relationship between the components of exercise health beliefs and physical activity and physical exercise behaviors. Future research designs will fully consider these indicators.

## Conclusion

This study revealed a significant negative correlation between perceived subjective barriers and exercise self-efficacy. To our knowledge, our study has provided a new psychological angle for understanding the exercise condition of college students and the restraining factors and offered new insights into increasing exercise self-efficacy, which is to lower the subjective barriers to exercise. To better explain the psychological mechanism of exercise health beliefs affecting college Students’ physical activity, future research designs will fully consider assessing the association between the components of exercise health beliefs, physical activity levels, and physical exercise behaviors.

## Data Availability Statement

The raw data supporting the conclusions of this article will be made available by the authors, without undue reservation.

## Ethics Statement

This study was approved by the Human Experimental Ethics Board of Sichuan University of Arts and Science (Approval no. 2022SASULL—001). The patients/participants provided their written informed consent to participate in this study.

## Author Contributions

LG and JS: conceptualization, investigation, resources, data curation, and writing—original draft preparation. LG: methodology. JS: writing—review and editing. Both authors have read and agreed to the published version of the manuscript.

## Conflict of Interest

The authors declare that the research was conducted in the absence of any commercial or financial relationships that could be construed as a potential conflict of interest.

## Publisher’s Note

All claims expressed in this article are solely those of the authors and do not necessarily represent those of their affiliated organizations, or those of the publisher, the editors and the reviewers. Any product that may be evaluated in this article, or claim that may be made by its manufacturer, is not guaranteed or endorsed by the publisher.
