# Corrigendum: The current health belief of exercise conditions of Chinese college students and ways of improvements: An analysis based on the health belief model

**DOI:** 10.3389/fpsyg.2023.1173887

**Published:** 2023-03-17

**Authors:** Lamei Gong, Jiazhi Sheng

**Affiliations:** ^1^Laboratory of Sports and Health Promotion, School of Physical Education, Sichuan University of Arts and Science, Dazhou, China; ^2^Graduate School of Management, Management and Science University, Shah Alam, Malaysia

**Keywords:** perceived benefits, perceived subjective barriers, perceived objective barriers, perceived severity, self-efficacy of exercise, cues to action

In the published article, there was an error in **Participants and Methods**, “*General Demographic Information Survey,”* Paragraph 1. The eighth option of the description of “family income” was written as “8.9000–1,000 (8 score)” but should be “8.9,000–10,000 (8 score)”. The corrected paragraph appears below.

The age, educational level of the participants, and their parents and family monthly income were included and were assigned points. A total of 1–4 points were assigned to educate under elementary education, high school or technical education, graduate degree, and master's degree. Points for family income (in RMB: Yuan/month): 1.2,000–2,999 (1 score), 2.3,000–3,999 (2 score), 3.4,000–4,999 (3 score), 4.5,000–5,999 (4 score), 5.6,000–6,999 (5 score) 6.7,000–7,999 (6 score), 7.8,000–8,999 (7 score), 8.9,000–10,000 (8 score), 9. more than 10,000 (9 score) (refer Table 1 for survey results).

In the published article, there was also an error in **Participants and Methods**, “*Questionnaire Design and Reliability and Validity Test,”* Paragraph 1. “Previous research elaborated that if the Cronbach's alpha coefficients of the subscale are more than 0.6” should have been “Previous research elaborated that if the Cronbach's alpha coefficients of the subscale are less than 0.6”. The corrected paragraph appears below.

According to experience, the first step is to examine the validity of the questionnaire. After inspection, the validity coefficient of the scale in this study was 0.821, and *P* < 0.001, which illustrated that the scale has good validity. Cronbach's alpha values were used for assessing the reliability of the scale. Previous research elaborated that if the Cronbach's alpha coefficients of the subscale are less than 0.6, it should be considered deleted (Bagozzi and Yi, 1988). Based on preliminary results, we found that the first item of perceived objective barriers, the third item of subjective barriers, and the fifth to fourteenth items of self-efficacy for exercise were deleted. Finally, the final version included 17 items loaded on six factors: perceived benefits (three items), perceived objective barriers (two items), perceived subjective barriers (two items), cues to action (three items), perceived severity (three items), and self-efficacy of exercise (four items).

In the published article, there was also an error in **Discussion**, “*Impact of Demographic Factors on Exercise Health Belief,”* Paragraph 1. “However, no such association not was found in our study” should have been “However, no such association was found in our study”. The corrected paragraph appears below.

Self-efficacy and perceived barriers were the important factors of exercise belief that impact exercise behavior. They were more than an essential topic for health education but a strong field of study in sports science. They can be considered a vital factor for predicting exercise behavior (Fletcher and Banasik, 2001). Previous studies believed that factors that impact self-efficacy included parents' education level (Fletcher and Banasik, 2001; Almutary and Tayyib, 2020) or their academic level and family income level (Sosa et al. 2021). Our study found a significant-association between monthly household income level and perceived self-efficacy, which is consistent with the study of Sosa et al. (2021); at the same time, Sosa et al. (2021) also described a significant positive-association between household income and perceived benefits. However, no such association was found in our study, possibly due to the different ages and cultural backgrounds of the participants. It implied that demographic/environmental factors contributed more to explaining physical activity level than the influential external factors. In addition, this study did not discover other factors that could influence the self-efficacy of exercise and health beliefs of Chinese college students in addition to the factors mentioned above. The probable reason could be that college students are independent in thoughts and behaviors, and the influence of family factors is reduced.

The authors apologize for these errors and state that this does not change the scientific conclusions of the article in any way. The original article has been updated.

